# Phylogeographic relationships and the evolutionary history of the *Carassius auratus* complex with a newly born homodiploid raw fish (2nNCRC)

**DOI:** 10.1186/s12864-022-08468-x

**Published:** 2022-03-28

**Authors:** Qianhong Gu, Shi Wang, Hui Zhong, Hui Yuan, Junliu Yang, Conghui Yang, Xuexue Huang, Xiaowei Xu, Yude Wang, Zehong Wei, Jing Wang, Shaojun Liu

**Affiliations:** State Key Laboratory of Developmental Biology of Freshwater Fish, College of Life Sciences, Engineering Research Center of Polyploid Fish Reproduction and Breeding of the State Education Ministry, Hunan Normal University, Changsha, 410081 Hunan P.R. China

**Keywords:** Eurasia, *Carassius auratus* complex, Homoploid hybrid speciation, Phylogenetics, Phylogeography

## Abstract

**Background:**

An important aspect of studying evolution is to understand how new species are formed and their uniqueness is maintained. Hybridization can lead to the formation of new species through reorganization of the adaptive system and significant changes in phenotype. Interestingly, eight stable strains of 2nNCRC derived from interspecies hybridization have been established in our laboratory. To examine the phylogeographical pattern of the widely distributed genus *Carassius* across Eurasia and investigate the possible homoploid hybrid origin of the *Carassius auratus* complex lineage in light of past climatic events, the mitochondrial genome (mtDNA) and one nuclear DNA were used to reconstruct the phylogenetic relationship between the *C. auratus* complex and 2nNCRC and to assess how demographic history, dispersal and barriers to gene flow have led to the current distribution of the *C. auratus* complex.

**Results:**

As expected, 2nNCRC had a very close relationship with the *C. auratus* complex and similar morphological characteristics to those of the *C. auratus* complex, which is genetically distinct from the other three species of *Carassius*. The estimation of divergence time and ancestral state demonstrated that the *C. auratus* complex possibly originated from the Yangtze River basin in China. There were seven sublineages of the *C. auratus* complex across Eurasia and at least four mtDNA lineages endemic to particular geographical regions in China. The primary colonization route from China to Mongolia and the Far East (Russia) occurred during the Late Pliocene, and the diversification of other sublineages of the *C. auratus* complex specifically coincided with the interglacial stage during the Early and Mid-Pleistocene in China.

**Conclusion:**

Our results support the origin of the *C. auratus* complex in China, and its wide distribution across Eurasia was mainly due to natural Pleistocene dispersal and recent anthropogenic translocation. The sympatric distribution of the ancestral area for both parents of 2nNCRC and the *C. auratus* complex, as well as the significant changes in the structure of pharyngeal teeth and morphological characteristics between 2nNCRC and its parents, imply that homoploid hybrid speciation (HHS) for *C. auratus* could likely have occurred in nature. The diversification pattern indicated an independent evolutionary history of the *C. auratus* complex, which was not separated from the most recent common ancestor of *C. carassius* or *C. cuvieri*. Considering that the paleoclimate oscillation and the development of an eastward-flowing drainage system during the Pliocene and Pleistocene in China provided an opportunity for hybridization between divergent lineages, the formation of 2nNCRC in our laboratory could be a good candidate for explaining the HHS of *C. auratus* in nature.

**Supplementary Information:**

The online version contains supplementary material available at 10.1186/s12864-022-08468-x.

## Background

A fundamental question in evolutionary biology is whether speciation is gradual or punctuated [1]. Hybridization is regarded as an ‘evolutionary catalyst’ that may be an important source for the origination of a new species or an increase the amount of genetic variability. Hybridization can result in the reorganization of adaptive systems and lead to the formation of new species [2]. Hybridization between species can trigger vast genetic and genomic imbalances, including a high rate of DNA mutations and combinations [3], often resulting in significant changes in phenotypes and genotypes of hybrid offspring, which may facilitate speciation and adaptive radiation.

Drastic climatic and geological oscillations, such as glacial expansion and megadroughts, forced freshwater species to contract their distribution ranges and reside in small refuges in many cases. Potential gene exchange and hybrid speciation likely occurred owing to the close contact between distant species. Increasingly, studies suggest that interspecies hybridization and gene introgression are not uncommon in fish, such as in the families Poeciliidae, Atherinidae, Cyprinidae, Cobitidae, and even Carcharhinidae [4–6], especially East African cichlids, which have attracted much attention in recent years [7–9]. As the most common species of vertebrates, more than 34,000 fish species have been recorded (https://www.fishbase.de/). The formation of many fishes has been determined to be associated with hybridization [10, 11]. Ancestral hybridization has been suggested to have played a key role in facilitating species diversification of cichlid fish in Lake Malawi, Lake Victoria, and Lake Tanganyika [7–9].

However, for a long time, there has been a lack of enough direct evidence to prove that fish hybridization will produce new species. Distant hybridization can generate allotetraploid and autotetraploid fish, supplying important and direct evidence for hybridization speciation in fish [12]. It was reported that a newly born homodiploid raw fish (2nNCRC, 2n =100), which is very similar to the crucian carp with no beard, was derived from interspecies hybridization between *Cyprinus carpio* (female, 2n = 100) and *Megalobrama amblycephala* (male, 2n = 48) by artificial propagation [13]. Fortunately, we repeated this experiment three times with similar results in our laboratory. In addition, eight stable generations of 2nNCRC with a very clear genetic background have been founded in our laboratory since 2014, supplying a valuable model system for studying hybrid speciation in fish.

It is widely believed that five species exist in the genus *Carassius*, including *Carassius carassius*, *Carassius cuvieri*, *Carassius langsdorfii*, *Carassius gibelio* and *Carassius auratus*, which are generally accepted as valid species and they can be found in FishBase (https://www.fishbase.de/). Our previous study showed that the hybrid speciation would be a possible route of formation of *C. auratus* [13]. It is particularly interesting to find out whether the origin of *C. auratus* is identical to that of 2nNCRC and whether *C. auratus* evolved from 2nNCRC. First and foremost, it is necessary to clarify the historical evolutionary processes of *C. auratus*. Phylogeographic studies might address this issue because they are usually used to determine the historical processes that affect vicariance, dispersal, extinction and radiation that then lead to the geographic distributions of genetic lineages [14, 15].

The recent climatic oscillation and geological events during the Pliocene-Pleistocene have played a fundamental role in shaping contemporary patterns of biodiversity and species diversification and distribution [16, 17]. Pleistocene glaciations are known to have had a far-reaching influence on the evolution of organisms in the Northern Hemisphere [18, 19]. For example, paleontological and genogeographical studies indicate that European and North American species experienced repeated episodes of contraction and expansion of their ranges due to major climatic oscillations [20]. The *Carassius* species complex has a wide distribution across the Eurasian continent and neighboring islands [21, 22]. According to the fossil record of *Carassius* species in the Pliocene epoch (5.3–2.6 million years ago, Mya) discovered in northern China [23], Quaternary paleoenvironmental changes in East Asia and Europe had a great influence on the speciation and diversification of *Carassius* species. In freshwater fishes, the dynamics of recolonization are tightly linked to the history of river drainage systems [24]. During glacial melt periods, ephemeral rivers and periglacial lakes could arise, and the reconfiguration of the landscape caused by drastic climatic change and geological events may have allowed species to disperse into new habitats, providing opportunities for colonization and new species interactions.

These processes have resulted in complicated recolonization scenarios for the *Carassius* species complex in East Asia, where the haplotypes of the mitochondrial control region and *tf* alleles are clustered into four and three major lineages, respectively, and based on this information, it has been speculated that the Yangtze River basin was the potential origination center for the *Carassius* species complex then radiated across East Asia [22, 25]. Gao et al. [21] deduced that the close relationship between the *C. auratus* complex from eastern mainland China and south-central Ryukyus was the result of natural Pleistocene dispersal. However, the existence of two distinct lineages of *C. carassius* in Europe [24] was mainly due to the Danubian catchment being separated from other river systems by the Alps, the Sudetes Mountains and the Carpathian Mountains.

According to the formation of the newly born 2nNCRC, combined with the close phylogenetic relationship between the *C. carpio* and *Carassius* species complex [26, 27], we speculated that the existence of a possible route through distant hybridization under natural conditions may have generated *C. auratus*, and this hybridization likely occurred during the Pliocene–Pleistocene based on the following hypotheses: (1) 2nNCRC has a very close relationship with the *C. auratus* complex in terms of phenotype and genotype; (2) several geographic lineages of *Carassius* are endemic to specific regions of Eurasia, especially the *C. auratus* complex in China; and (3) the most recent common ancestor (TMRCA) of both *Cyprinus* and *Megalobrama* had a much earlier divergence time than that of the *C. auratus* complex, and all had a sympatric distribution in history. According to the distribution of current fish species and fossil data [23, 28], the speciation of *C. auratus* in China might have been punctuated and not derived from the processing of lineage differentiation with other species of *Carassius*. Thus, establishing a robust time-calibrated phylogeny is the first requirement for tracing the possible origin and diversification patterns of the *Carassius* species complex.

In the present study, the mitochondrial genome and nuclear DNA were used to reconstruct the phylogenetic relationship between 2nNCRC and the *C. auratus* complex. The cytb was the largest data set including the Genbank data, including the population information for the species of *Carassius*, were obtained and used to investigate the evolutionary history of the *C. auratus* complex.

## Results

### Morphological traits

**Fig. 1:**
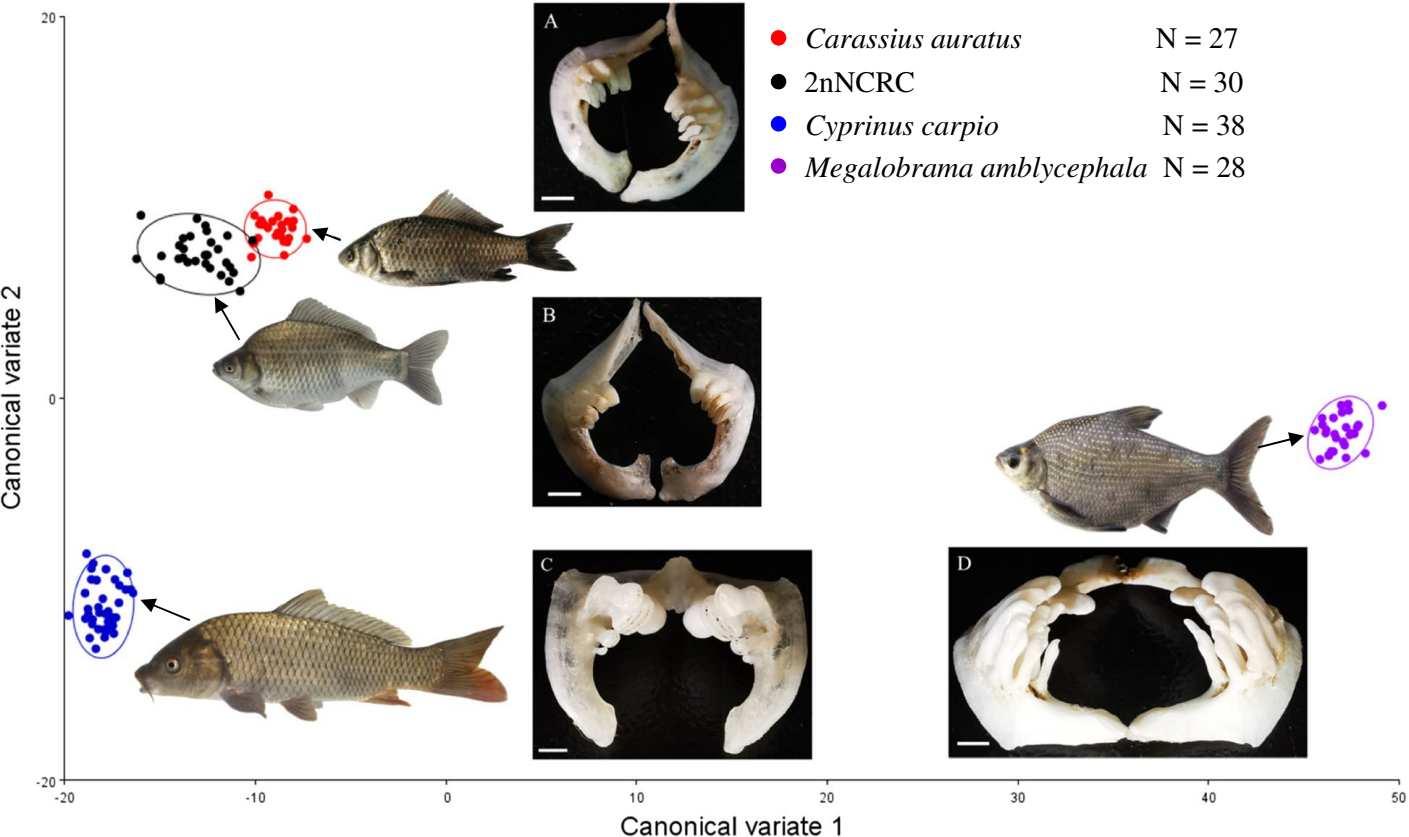
Scatter plots from the Canonical Variate Analysis (CVA) for *C. auratus*, 2nNCRC, *C. carpio* and *M. amblycephala*, and number (N) of each species was shown. **A** The pharyngeal teeth of *C. auratus* possessed four compressed teeth in each side. Bar = 0.25 cm. **B** The pharyngeal teeth of 2nNCRC same as (**A**). Bar = 0.25 cm (**C**) The pharyngeal teeth of *C. carpio* possessed rounded and ridged molariform teeth. Bar = 0.28 cm. **D** The pharyngeal teeth of *M. amblycephala* possesses recurved and uncinate teeth. Bar = 0.33 cm

Twelve to twenty samples of each generation of 2nNCRC were randomly selected to summarize the morphological characteristics of qualitative and quantitative traits (Table S1 in Supplementary Information). Both 2nNCRC and *C. auratus* have no barbells and almost the same quantitative traits. In particular, they have the same morphotype and pattern of pharyngeal teeth and four tabular teeth on each side of the pharyngeal bone, which are very different from those of *C. carpio* and *M. amblycephala* (Fig. 1). 2nNCRC (*n* = 30), *C. auratus* (*n* = 27), *C. carpio* (*n* = 38) and *M. amblycephala* (*n* = 28) were used to examine shape variation through principal component analysis (PCA) and canonical variance analysis (CVA). The results showed that the first three PCs explained 94.13% of the variation in shape morphology (Table S2 in Supplementary Information), while the first two CVs explained 97.94% of the variation (Table S3 in Supplementary Information). The scatter plot of CV1 and CV2 showed clear differentiation between 2nNCRC and its parents (Fig. 1) but a close relationship and partial overlap between 2nNCRC and *C. auratus*. The Mahalanobis distances based on geometrical morphology data showed a significant difference among 2nNCRC and the three species (*P* < 0.0001) (Table S4 in Supplementary Information), while the distance values between 2nNCRC and *C. auratus* were much lower than those between 2nNCRC and its parents.

### Phylogenetic tree and haplotype network

**Fig. 2:**
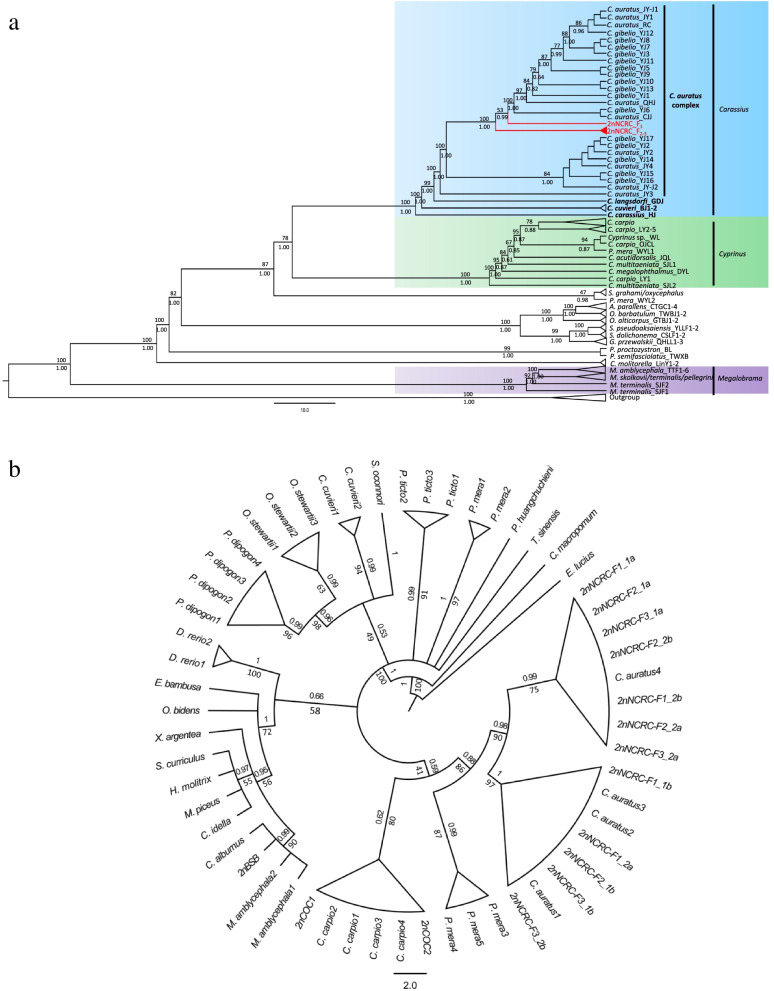
**a** ML and Bayesian tree of *Carassius* with 2nNCRC and some representative Cyprinidae fish (including 29 specimens of *Carassius*, 19 of *Cyprinus*, 14 of *Megalobrama* and 23 of other cyprinid fish) using complete mitochondrial genomes based on the concatenated dataset (twelve proteins and two rRNAs); **b** ML and Bayesian tree of 2nNCRC and Cyprinidae (40 specimens representing 21 species) using the partial cds of HoxA2b. Numbers at nodes are maximum likelihood bootstrap values (up the branch) and Bayesian posterior probabilities (down the branch)

The aligned and concatenated sequences (14 mitochondrial genes) were 13,634 bp with 5841 variable sites. Of these, 816 were parsimony-informative. The maximum likelihood (ML) and Bayesian tree showed a clear identification of the species of *Carassius* and most species of Cyprinidae (Fig. 2a). All species from *Carassius* containing 2nNCRC formed a monophyletic clade with high support values, and three valid species of *Carassius* were observed (Fig. 2a, in bold). However, 2nNCRC, together with *C. gibelio*, was unable to be separated from *C. auratus* but formed a single genetic cluster, and the genetic distances among 2nNCRC, *C. auratus* and *C. gibelio* were much lower than those among 2nNCRC and other species of *Carassius* (Table S5 in Supplementary Information). Consequently, *C. auratus* and *C. gibelio* are named the *C. auratus* complex in the present study.

Another phylogenetic tree using HoxA2b (nuclear DNA) also clearly identified most species of Cyprinidae and two distinct clusters in the *C. auratus* complex with 2nNCRC, which were monophyletic and sister to *Procypris merus* and *C. carpio*. However, it was also impossible to distinguish between 2nNCRC and *C. auratus* (Fig. 2b).

**Fig. 3:**
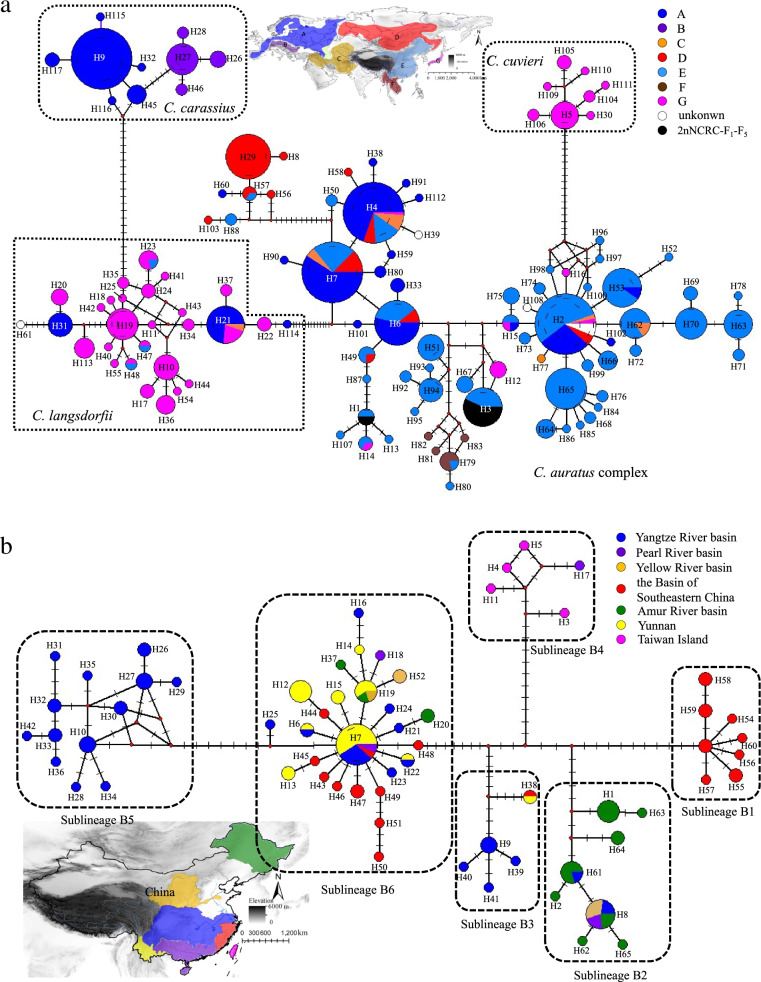
Median-joining haplotype network based on a) 831 sequences of cytb including 15 2nNCRC and 816 specimens of *Carassius* (see Additional file 3 in Supplementary Information) derived from GenBank, where the specimens of *Carassius* in the network were found: **A** the Europe, except for the south of Alpes mountains and Danube River basin, **B** the south of Alpes mountains and Danube River basin in Europe, **C** Western Asia, **D** Siberia in Russia and Mongolia, **E** China, **F** Southeast Asia, **G** the Japan and Ryukyu Islands; and b) 128 sequences of cytb of *C. auratus* complex just distributed in China. The size of the circles represents haplotype frequency. Each connecting line represents a single nucleotide substitution, and each little short line represents mutated position. The haplotypes of different geographic samples were represented as different colors

The median joining network of cytb showed that there was no shared haplotype among the *C. carassius*, *C. cuvieri*, *C. langsdorfii* and *C. auratus* complexes (Fig. 3a). The haplotype of *C. carassius* was distributed in Europe with two distinct sublineages. The haplotype of *C. cuvieri* had a narrow distribution in Japan, and most of the haplotypes of *C. langsdorfii* were distributed in Japan with very few shared haplotypes (Fig. 3a). In addition, there were two shared haplotypes (H1 and H3) between 2nNCRC and *C. auratus*. However, the *C. auratus* complex had an admixture distribution across Eurasia with many shared haplotypes, such as H2, H4, H6 and H7, which could be found in Europe, China, Western Asia and Siberia (Fig. 3a). Surprisingly, a significant phylogeographical pattern with four specific regions was found for the *C. auratus* complex in China, two in Southeast China (Sublineage B1 in Fujian Province, Sublineage B4 in Taiwan Island), one in North China (Sublineage B2 in Amur River Basin), and one in the middle of the Yangtze River basin (Sublineage B5) (Fig. 3b).

### Divergence time estimation

**Fig. 4:**
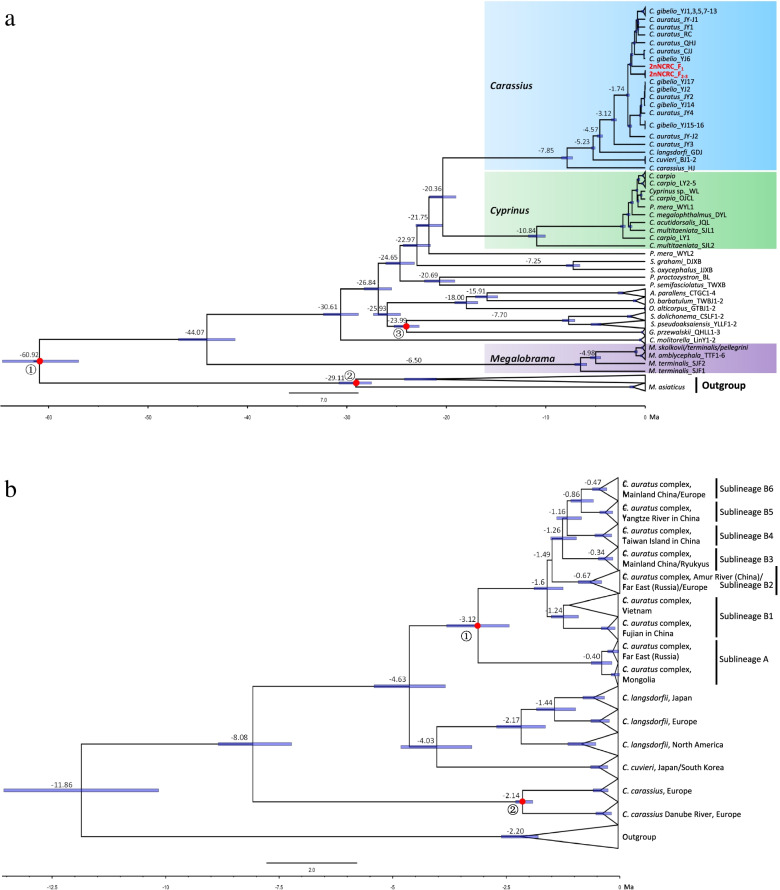
**a** Dated phylogeny using the concatenated dataset (twelve proteins and two rRNAs) with three calibration points (red dots indicate time-calibration markers, ①: Cavender, 1986 [31]; ②: Liu and Chang, 2009 [32]; ③: Harrison, 1992 [33]), and **b** using cytb gene with two calibration points (red dots indicate time-calibration markers, ①: Liu and Su, 1962 [23]; ②: Jeffries et al., 2016 [24]) to estimate the divergence of *Carassius*. Node ages are shown together with 95% highest posterior density bars indicating a range of age estimates

The fossil-calibrated molecular clock using the 14 incorporated genes from mitochondria showed that most clades were estimated to approach the earliest known fossils in time; for example, the TMRCA of Cyprinidae in the present study was approximately 44.07 Mya (Fig. 4a), close to the oldest and reliable fossils of Cyprinidae that are from the mid-Eocene (48.6 Mya, Fig. 4a) (Patterson, 1993). TMRCA of Schizothoracinae was dated at approximately 23.99 Mya (Fig. 4a), coinciding with the origin time of schizothoracine fishes estimated by Ruber et al. [29] and Wang et al. [30], in the Oligocene-Miocene boundary (approximately 23 Ma, Fig. 4a). The divergence between *Cyprinus* and *Carassius* was approximately 20.36 Mya (95% HPD: 21.69–19.04 Mya) (Fig. 4a). TMRCA of *Megalobrama* and *Cyprinus* was dated 6.5 Mya (95% HPD: 7.08–6.88 Mya) and 10.84 Mya (95% HPD: 11.68–9.98 Mya), respectively (Fig. 4a). Both of these values were much earlier than that of the *C. auratus* complex (3.12 Mya, Fig. 4a, b). The molecular data estimated by the incorporated 14 genes and by cytb only yielded similar results for the first divergence of *C. carassius* from the *Carassius*, at approximately 7.85 Mya (95% HPD: 8.43–7.30 Mya, Fig. 4a) and 8.08 Mya (95% HPD: 8.84–7.23 Mya, Fig. 4b), respectively. They both dated TMRCA of the *C. auratus* complex during the Late Pliocene (at approximately 3.12 Mya) with clear consistency, although the approaches used different datasets and fossil calibrations. However, there also existed a subtle difference between the two dates *C. cuvier* was separated at approximately 5.23 Mya (95% HPD: 5.42–5.04 Mya, Fig. 4a) using the 14 incorporated genes, while the TMRCA of *C. cuvier* and *C. langsdorfi* dated to approximately 4.67 Mya (95% HPD: 5.41–3.84 Mya, Fig. 4b) using only cytb; in addition, *C. cuvier* split from *C. langsdorfi* at approximately 4.03 Mya (95% HPD: 4.82–3.26 Mya, Fig. 4b).

Within the widely distributed *C. auratus* complex, several sublineages were found in Eurasia, and most were consistent with the six sublineages (sublineages B1-B6) distributed in China, except for sublineage A from the Far East (Russia) and Mongolia, which first diverged from the *C. auratus* complex at approximately 3.12 Mya (95% HPD: 3.81–2.44 Mya, Fig. 4b). Sublineage B1 from Fujian in China and Vietnam was estimated to have separated at approximately 1.6 Mya (95% HPD: 1.89–1.25 Mya, Fig. 4b), followed shortly thereafter by the divergence of sublineage B2, which was mostly distributed in the Amur River basin and Eurasia at approximately 1.49 Mya. Sublineage B3 was distributed in mainland China and Ryukyus and split at approximately 1.26 Mya. Two specific sublineages, B4 (on Taiwan Island) and B5 (in the Yangtze River basin), separated from the others at approximately 1.16 Mya (95% HPD: 1.39–0.85 Mya, Fig. 4b) and 0.86 Mya (95% HPD: 1.08–0.59 Mya, Fig. 4b), respectively. Sublineage B6 (widely distributed in mainland China and Europe) diverged at approximately 0.47 Mya (Fig. 4b).

**Fig. 5:**
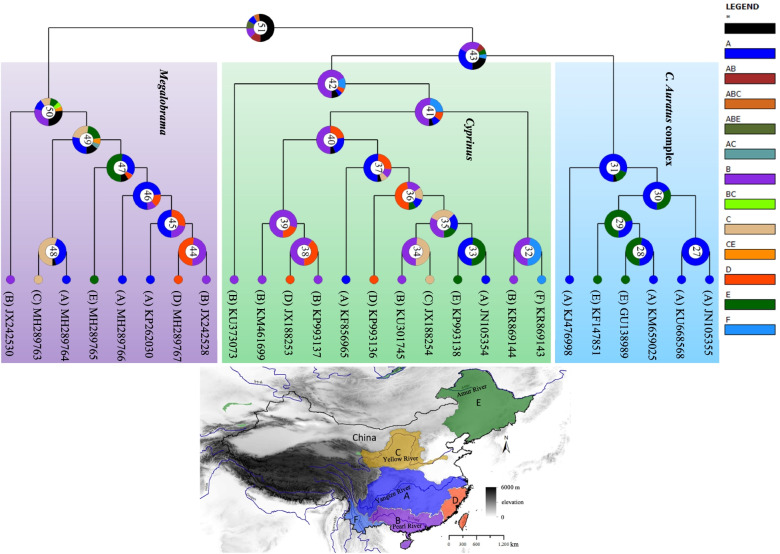
Ancestral range reconstruction for *C. auratus* complex, *Cyprinus* and *Megalobrama* just distributed in China using 26 mtDNA. The colors of the charts correspond to the most likely ancestral areas inferred, and the black color means the unknown area. Letters represent the biogeographic regions for *C. auratus* complex: **A** the Yangtze River basin, **B** The Pearl River basin, **C** the Yellow River basin, **D** the River Basins of Southeastern China, **E** the Amur River basin, **F** River Basins of Southwestern China. The blue curves in the distribution map of *Carassius* mean the river systems

### Ancestral range reconstruction

The results of the ancestral range reconstruction are depicted in Fig. 5 and Fig. S2 (in Supplementary Information). Among the six models of geographic-range evolution compared in the likelihood framework in Bio-GeoBEARS, the DIVA + *j* model was chosen for both 24 cytb sequences and 26 concatenated dataset (twelve proteins and two rRNAs in mitogenomes) according to the best likelihood and AICc associated scores (Table S6 in Supplementary Information). According to the ancestral area reconstruction using the 24 cytb gene across Eurasia, 41 dispersal and 21 vicariance events occurred within the evolution of the studied *Carassius*. TMRCA of *Carassius* probably diverged into two distinct clades (Fig. S2) in the late Miocene. One clade included *C. carassius*, which was mainly distributed in Europe, while the other clade further split into two separate lineages through two dispersal events and one vicariance event; the dispersal events corresponding to TMRCA of *C. cuvieri*, which was likely to disperse from Eurasia during early Pliocene according to the molecular dating (Fig. 4b). And then it diverged in two lineages through a vicariance event, the first one corresponding to lineages distributed in Japan (*C. cuvieri* and *C. langsdorfii*, node 43, G: 65.86%), and the second one corresponding to the lineage of *C. auratus* complex, whose ancestor was most likely to live in China (node 40). Three vicariance events occurred within the first lineage: a first one about 4.03 Ma separating *C. cuvieri* from *C. langsdorfii*, and other events during Pleistocene leading to different sublineages of *C. langsdorfii* across Eurasia. Within the second lineage, fifteen diversification events occurred: a vicariance event between Far East (Sulineage A) and China (Sublineage B) about 3.12 Mya, and fourteen events between 1.6–0.34 Mya leading to sublineages B1–B6. The dispersal events from the China to the Southeast Asia (Vietnam) explain the actual distribution of Sublineage B1 in both areas. The same condition can be found for the Sublineage B2 co-occur in the North China (Amur River Basin) and Far East, as well as the Sublineage B3 co-occur in China and Ryukyus and Japanese Islands. According to the ancestral area reconstruction using the 26 concatenated dataset of the *C. auratus* complex, *Cyprinus* and *Megalobrama* were distributed in China, and both TMRCAs of *Cyprinus* (node 42) and *Megalobrama* (node 50) were mainly distributed in the Pearl River basin, while TMRCA of the *C. auratus* complex was mainly distributed in the Yangtze River basin (node 31, A: 80.28%) (Fig. 5).

## Discussion

This study represents the first attempt to better understand the relationship of artificial hybrid fish species and naturally evolving fish. 2nNCRC is very similar to *C. auratus* in terms of morphological characteristics based on qualitative and quantitative traits, as well as the geometric shape of fish. Our phylogenetic analysis showed that *C. auratus* and *C. gibelio* were unable to be separated from each other based on mtDNA and HoxA2b but were distinct from the other three species of *Carassius*. The intraspecies relationship between 2nNCRC and *C. auratus* complex was uncovered by both mtDNA and HoxA2b. The *C. auratus* complex was likely to have originated in China, and the admixture of mtDNA haplotype lineages across Eurasia was mainly due to natural Pleistocene dispersal and/or anthropogenic translocation.

### Prerequisite conditions and ecological selection for the HHS of 2nNCRC

2nNCRC has two sets of chromosomes, one each from the maternal *C. carpio*, and the great morphologic and genetic variation in the parents can be seen as HHS [13]. Compared with allopolyploid hybrid speciation, HHS has at least two issues. First, reproductive isolation can easily be generated between the allopolyploid hybrid species and their parents due to the inaccuracy of chromosomal pairing and the inability to formulate germ cells [34], while reproductive isolation cannot be immediately generated between HHS and parents, such as 2nNCRC and *C. carpio*. Second, two complete genomes from their parents can be found in allopolyploid hybrid species; they possess a protective mechanism for the genome, while HHS lacks this mechanism. However, the F_1_ 2nNCRC females and males are fertile, and eight stable strains of the new homoploid species have been established in our laboratory since 2014, suggesting that HHS not only is common in plants but also occurs in fish.

The prerequisite condition for HHS is that the distribution of parents should overlap in history, making hybrid speciation possible [35]. Both TMRCAs of *Cyprinus* and *Megalobrama* had a sympatric distribution in the Pearl River basin and Yangtze River basin (Fig. 5), and currently, they have a wide distribution with the *C. auratus* complex in these river systems. In particular, the connectivity between the Yangtze River and Pearl River system before the early Pleistocene would have facilitated the dispersion of freshwater species between the two river systems [36, 37]. Consequently, the connectivity of river systems and flooding mediated dispersion and encounters among freshwater species.

In addition, occupying a different and suitable ecological niche from parents is very important for HHS [7, 37], and this scenario which may be lost during continuous backcrossing with parents. Fortunately, the reproductive trait of 2nNCRC was the same as that of the *C. auratus* complex, both reaching sexual maturity at the age of 1 year [13]. *C. carpio* and *M. amblycephala* reach sexual maturity at least 2 years old. Furthermore, the same shape and dental pattern of pharyngeal teeth were found for 2nNCRC and the *C. auratus* complex (Fig. 1). The same compressed teeth in *C. auratus* and 2nNCRC suggested that they had the same omnivorous feeding habit but mainly fed on plant food (the stem and leaf of aquatic plants). However, rounded and ridged molariform teeth are found in *C. carpio*, which feeds on omnivores but mainly on animal food (freshwater snails and aquatic insects). *M. amblycephala* possesses recurved and uncinate teeth and mainly feeds on aquatic plants. In terms of freshwater aquatic resources, in comparison to *C. carpio* and *M. amblycephala*, the homoploid 2nNCRC and *C. auratus* have stronger adaptability, as they have a wider range of food in the aquatic ecosystem. Ecological selection is a major factor promoting HHS, and the foundation of a novel ecological niche supplies an opportunity for the evolution of new species while maintaining its own unique phenotype [37]. The family Cyprinidae exhibits species-specific numbers and arrangements of pharyngeal teeth, which are an important feeding organ and taxonomic characteristic, and extensive variation in tooth shape with high hereditary stability [38]. The same pharyngeal teeth were found among the *C. auratus* complex and different generations of 2nNCRC. Although the homoploid 2nNCRC and *C. auratus* showed very similar phenotypes, there were also significant differences between them, including the qualitative and quantitative traits found by Wang et al. [13] and the geometrical morphological shapes, which exhibited a significant humped back in 2nNCRC, found in the present study (Fig. 1). The morphological variation between 2nNCRC and *C. auratus* might be attributed to long-term morphological evolution.

### Evolutionary relationships of the *C. auratus* complex with the homoploid 2nNCRC

In the phylogenetic tree, ML and Bayesian analyses based on the concatenated sequence produced essentially the same tree topology with high ML bootstrap values and Bayesian posterior probabilities. We determined that the robust tree topology adequately reflected the phylogeny of the species in *Carassius* and *Cyprinus*, as well as other species in Cyprinidae. *Carassius* clustered with *Cyprinus* first and then clustered with other species in Cyprinidae, Barbinae, Schizothoracinae and Labeoninae, all of which belong to Barbini, a monophyletic group in Cyprinidae, and this result was consistent with those of previous phylogenetic studies and the morphological characteristics of Cyprinidae [39, 40]. *Carassius*, as a monophyletic clade, included four definite interspecies clusters, and there was no shared haplotype among *C. carassius*, *C. cuvieri*, *C. langsdorfii* and other species, including *C. gibelio* and *C. auratus* (Fig. 3a). The haplotypes of *C. carassius* observed in Europe were separated into two clades without shared haplotypes, in accordance with the existence of two *C. carassius* lineages in Europe [24]. The haplotypes of *C. cuvieri* were restricted to mainland Japan, and the major haplotypes of *C. langsdorfii* observed across Eurasia were distributed on Japanese islands. Considering that the distribution of haplotypes was restricted to specific areas, we considered that the three species evolved independently for a considerable period of time on the main Japanese islands and Europe. However, the widely distributed species *C. gibelio* and *C. auratus* were paraphyletic, and they had many shared haplotypes. It has been documented that the triploid *C. gibelio* has a more shared haplotype with the sympatric diploid *C. auratus* than with allopatric triploids [41]. Previous studies have proposed that an autopolyploidy event might have resulted in the formation of *C. gibelio* (3n = 150) [42, 43]. Liu et al. [22] considered that the polyploid *C. gibelio* might have originated from the sympatric *C. auratus* (2n = 100) via autopolyploidy. However, it was reported that the autopolyploidy has not contributed to the separation between diploid *C. auratus* and triploid *C. gibelio* as they were both intermixed in the same lineages and share the same alleles [22, 25]. Although *C. gibelio* is naturally gynogenic, many phenomena similar to those in normal sexual diploid species, such as normal meiosis, multiple modes of unisexual gynogenesis and sexual reproduction, have been identified in some strains of *C. gibelio* [44, 45]. Hence, there exist the possibility of the breeding between diploid *C. auratus* and triploid *C. gibelio* in theory. Furthermore, the shared haplotype between the sympatric *C. gibelio* and *C. auratus* found at present and in previous studies confirmed the existence of gene flow between them. Consequently, it is likely that extensive gene flow and no substantial genetic separation exist between *C. gibelio* and *C. auratus*. Though the lack of genetic separation between them could be also the result of recent divergence, TMRCA of *C. auratus* complex and divergence between sublineage A and B, as well as among B1-B6 could be traced back to as early as the later Pliocene (Fig. 4b) indicating a complex evolutionary history for *C. auratus* complex.

The preliminary analysis of the nuclear genome of the homoploid 2nNCRC suggested that no characteristic exists that distinguishes the homoploid 2nNCRC from the diploid *C. auratus* [13]. The homoploid 2nNCRC was clustered with *C. auratus* based on mtDNA and nuclear DNA (HoxA2b) data. However, the genus *Carassius* was not a monophyletic group in the HoxA2b tree and showed a closer relationship between the *C. auratus* complex and *C. carpio* (Fig. 2b). The nuclear–mitochondrial discordance in the phylogenetic placement of *C. auratus* and *C. cuvieri* suggested a possible complex evolutionary history of the genus *Carassius*. Certainly, more nuclear markers and specimens are needed to further investigate the phylogenetic relationship in *Carassius*, and further studies including genomic data are required. We found six sublineages, and four had high regional specificity that were composed of endemic populations indigenous to each region in China (Fig. 3b), including Southeast China (Fujian), North China (Aumer River basin), Taiwan Island, and the middle and lower Yangtze River basin. However, a perplexing but concurrent event occurred in which the *C. auratus* complex found in Japan was not related to the Japanese crucian carp (*C. langsdorfii* or *C. cuvieri*) and the *C. auratus* complex widely distributed in Europe was not related to European crucian carp (*C. carassius*). The *C. cuvieri* was the monophyletic group (Fig. 2a) and was native to Japan [21]. However, the *C. auratus* complex in Japan was clustered with Chinese lineages. Similarily, most of the *C. auratus* complex across Europe examined in this study was nested in native Chinese lineages, and there were many shared haplotypes between Europe and China (Figs. 3a, 4b). The *C. auratus* complex distributed in Europe possibly originated via artificial introduction from Asia [41]. In contrast, genealogical analyses clustered the haplotype of *C. gibelio* distributed in Far East Russia (H29) with strong support, as well as two haplotypes with typical regions (H12 and H16) found in Ryukyus (Fig. 3a) and three specific haplotypes (H81, H82 and H83) found in Vietnam, suggesting that the *C. auratus* complex rapidly radiated from mainland China rather being distributed through recent anthropogenic translocation [41].

### Biogeographic history of the *C. auratus* complex

Patterns of divergence suggest historical biogeographic events. Sublineage A from Far East Russia and Mongolia first split from the *C. auratus* complex at approximately 3.12 Mya (Fig. 3b), far preceding the Pleistocene glacial epoch in China, suggesting that dispersal from China rather than vicariance created the current distribution pattern of the *C. auratus* complex in Asia (Fig. 4a). With the humid and warm climate during the early Pliocene in North China [46], which supplied a suitable habitat for freshwater species, the well-dated *Carassius* fossil in North China has been recorded in the Pliocene epoch. Furthermore, recurrent glaciations during the Pleistocene were another contributor to species diversification in East Asia. The early separation of sublineage B1 at approximately 1.6 Mya exactly coincided with the Poyang Glaciation (1.8 ~ 1.5 Mya) in China [47, 48] and Gonzi Glaciation in Europe. Sublineage B1 only existed in warm and moist tropical or subtropical areas (Vietnam and Fujian, China), which provided important refugia for freshwater species during glaciations [21, 49]. Many haplotypes found in sublineage B2 and sublineage A were *C. gibelio*, while sublineage B2 has a closer genealogical relationship with the *C. auratus* complex in China; the separation of sublineage B2 in the Amur River basin of China occurred at approximately 1.49 Mya, far postdating the divergence of sublineage A, suggesting that sublineage B2 was the result of another dispersal event during the Poyang-Dagu interglacial stage (1.5–1.1 Mya) [47, 48]. Furthermore, during interglacial times, the warm, wet East Asian summer monsoons intensified, which also promoted the dispersion of the *C. auratus* complex from China to Ryukyus [21]. In sublineage B3, the divergence between the *C. auratus* complex in Ryukyus and China dated to approximately 0.84 Mya, exactly coinciding with the Poyang-Dagu interglacial stage (0.9 ~ 0.4 Mya) in the Mid-Pleistocene [47, 48], and this result was consistent with our previous speculation that natural dispersed from mainland China to Ryukyus occurred in the Pleistocene. Sublineages B3 and B6 distributed across mainland China may be the result of repeated episodes of range contraction and expansion during glacial cycling. Most of the eastward river systems in mainland China are known to have repeatedly anastomosed with one another during the postglacial and Holocene due to erosion and stream capture [50], especially with recent anthropogenic translocations. In turn, these events likely had a dramatic effect on the evolution of many freshwater species, and might have promoted genetic exchange between previously isolated *C. auratus* complex lineages and even removed any geographic signatures. A similar situation can be found in other aquatic organisms, such as freshwater snails and frogs [49, 51]. However, a specific sublineage, B5, was found in the middle and lower Yangtze River basin, as well as three other sublineages (B1, B2 and B4) in China with specific regions, suggesting that the genetic structure in the *C. auratus* complex has not been completely changed by recent human-mediated translocations [21].

### *C. auratus* complex not a lineage differentiated from the common ancestor of *C. cuvieri* or *C. carassius*

Combined with the reconstruction of ancestral state and haplotype networks in the present study and a previous study on the population genetics of the *C. auratus* complex in China [21, 25], we concluded that the *C. auratus* complex originated in the Yangtze River basin in China (Fig. 5). TMRCA of *C. carassius* was mostly distributed in Europe, and both *C. langsdorfii* and *C. cuvieri* were on the main islands of Japan, essentially correspond to the results of a previous report on the distribution of some lineages of *Carassius* endemic to particular geographical regions [52–56].

It was believed that the freshwater ichthyofaunas from the islands of Japan and China resembled each other during the Miocene and Pliocene, as the main islands of Japan and mainland China are known to have formed a contiguous land mass in the late Pliocene [57]. Fossils indicate that in the late Pliocene, *C. auratus* was distributed in North China and Japan [23, 58], coinciding with the dating of TMRCA of the *C. auratus* complex in this study. Thus, we hypothesized that the *C. auratus* complex lineage should be separated from the *C. cuvieri* lineage based on molecular data and that a recent common ancestor existed for those distributed in Asia. The divergent and current distribution patterns of *Carassius* were mainly caused by dispersal that was aided by the formation of land bridges between Japanese archipelago islands and the continent [59, 60]. The common ancestor of the *C. cuvieri* and *C. auratus* complex likely dispersed from the Eurasian continent to the islands of Japan rather than in the reverse direction. Consequently, TMRCAs of *C. cuvieri* and *C. auratus* should be distributed on mainland China, which is incompatible with the present study (Fig. S2, node 44). Hence, there are reasons to believe that *C. auratus* did not diverge from TMRCA of *C. cuvieri*.

Similarly, TMRCA of *C. carassius* was distributed in Europe. However, the *C. auratus* complex currently distributed in Europe has been verified as dispersing from Asia by anthropogenic translocation. For instance, recent genetic evidence supports the anthropogenic introduction of the crucian carp to the UK during the fifteenth century [61]. *C. carassius* is native to parts of central, eastern and northern Europe and almost exclusively restricted to lentic ecosystems [56, 62]. A strong geographic structure was found because of the susceptibility of *C. carassius* to genetic isolation and bottlenecks due to their small population sizes and especially their low dispersal rates [24]. Two genetically distinct sublineages of *C. carassius* distributed in Europe were found in the present study and in a previous study [24]; therefore, the Carpathian Mountains and the Central European Highlands are inferred to have acted as barriers to the colonization of *C. carassius* in northern European drainages during the Pleistocene. According to the reconstructed ancestral areas, *C. carassius* was mainly distributed in Europe and restricted to its native areas (Fig. S2). Consequently, very few records of *C. carassius* distributed in Asia were found, except in West Siberia, Kazakhstan, Uzbekistan and Turkey, all of which are very close to Europe with no significant geographical barrier with Europe. However, the Central Siberian Plateau, Mongolian Plateau, Altay Mountains, Tianshan Mountains, Pamir Mountains, Himalayas, Iran Plateau and Great Caucasus, with elevations greater than 1000 m, formed natural dispersal barriers in the watersheds impeding freshwater species exchange. Furthermore, the separation of *C. carassius* from other species of *Carassius* far postdated the formation of high-elevation orogens such as the Tibetan Plateau, Mongolian Plateau and Tian-Shan, which occurred during the Miocene and even earlier [63]; in addition, well-dated *Carassius* fossils have been found in North China [23] that have far postdated the formation of these mountains, suggesting that *C. auratus* is not a lineage that diverged from TMRCA of *C. carassius*.

### Paleoclimate and geological events facilitated distant hybridization

Based on the above analysis and the homoploid 2nNCRC derived from distant hybridization in our laboratory [13], our results also reinforce a possible naturally occurring hybrid origin of *C. auratus*. Rapid hybrid speciation was recently shown to occur by Lamichhaney et al. [64], and many species of cichlid fishes in East Africa have been verified as experiencing ancestral distant hybridization that has been suggested to play a key role in facilitating species diversification [7–9]. Our findings further supported that this mechanism was very likely to have occurred in the Cyprinidae family.

The robust, time-calibrated molecular phylogeny suggested that the separation of *C. carpio* and *Carassius* occurred at approximately 11.86 Mya (Fig. 4b), close to the divergence between goldfish and common carp (10.0 Mya) [65], and the origin of the common ancestor of common carp and crucian carp (11.4–8.1 Mya) [66], all of which occurred close to the time of the hybrid speciation of allotetraploid *C. carpio* (12.4 Mya) as estimated by Xu et al. [27]. Our results showed that extreme climate oscillations and geological events that occurred during the Pliocene and Pleistocene might have had a great influence on the speciation and radiation of the *C. auratus* complex in China.

Landscape features and geological history are hypothesized to strongly affect biogeographical processes, and this can be especially true in freshwater systems [67, 68]. One major consequence of extreme climatic changes and geological activities is ecosystem and landscape reconfigurations. A specifically large event was the drastic uplift of the Tibetan Plateau that which ultimately led to the formation of eastward-flowing drainage systems in China, such as the Yangtze River, which formed between the Oligocene and Miocene, but the unification of the upper and middle Yangtze River in the Three Gorges Mountain region occurred between the Late Pliocene and Early Pleistocene [69, 70]. The Yangtze River and Han River flowed into the middle of the Jianghan basin. The connection of the upper and middle Yangtze River facilitated species dispersal from west to east, similar to what was observed for the frog *Quasipaa boulengeri* [49] and the freshwater snail *Bellamya aeruginosa* [51, 71]. Therefore, the establishment of the eastward-flowing Yangtze River and the changes in river systems could have provided opportunities for dispersal, gene flow and even hybridization between closely related freshwater species, especially fish. Furthermore, the intensified tectonic movement and glacial cycling in the Pleistocene, as well as the prevailing monsoon, had major impacts on East Asian biota starting in the late Pliocene [72, 73]. As a result, the weather was cold and dry, and rivers and lakes experienced frequent range expansions and regressions in the Jianghan and Dongting Lake basins [74, 75]. The regression of lakes and rivers could dramatically reduce the habitats available to fish, and this precarious situation was further exacerbated in the glacial period. Consequently, restricted habitat regions caused hybridization between close and even divergent lineages. An example of a specifically large event is in the LGM, where the sea level was 130–150 m lower than that today, and this event resulted in strengthening downcutting processes of rivers. Most lakes in the plains region of eastern China have opened and dried, and this scenario has been verified in Taihu Lake, Boyang Lake and Dongting Lake [76]. The cooler and drier climates intensified during this period, and the fish habitats in these lakes were fragmented or even lost. Hybridization between divergent lineages would have been common when habitats were repeatedly lost, as the propagation of vast majority of freshwater fishes is oviparity, creating opportunities for new speciation and diversification, especially for sympatric species possessing similar ecological and breeding habits, such as the parents of 2nNCRC in the Yangtze River basin. Both *C. carpio* and *M. amblycephala* with sympatric distribution in history (Fig. 5) mostly occupy the middle and lower layers in freshwater, and they usually breed during May–June in every year [77]. A typical example of this scenario is the repeated range expansions and regressions of lakes that likely contributed to the high diversity of African cichlids [78, 79]. Since East Africa underwent dramatic climatic and geological changes in the Pleistocene over the past few million years, the constant expansion and regression of the great East African lakes have led to the repeated loss of habitats or the formation of new habitats [51, 79]. For example, hybridization might have facilitated speciation bursts for the cichlids in Lake Tanganyika, and time-calibrated trees support the concept that the radiation of Tanganyika cichlids coincided with lake formation [8]. Specially, the ancient hybridization of Lake Victoria cichlids is considered to be related to the capture of Malagarasi (Congo) tributaries and East African mega-droughts, providing an opportunity for the Congolese lineage to colonize the Lake Victoria region and the Congo-Nilotic admixture event to occur [7]. It has been speculated that hybridization between distant relatives, when coincident with ecological opportunity, may facilitate rapid adaptive radiation through recombination and sorting of admixture-derived polymorphisms by natural and sexual selection [7]. The *C. auratus* complex exhibits remarkable morphological and genetic diversity, and the different ploidy forms, unisexual gynogenesis and bisexual reproduction [21, 80, 81], might also benefit from hybridization-derived genetic variants. Future investigation will test for evidence of ancient admixture among the *C. auratus* complex, *C. carpio*, and *M. amblycephala* based on whole-genome sequencing data and the effect of ancestral hybridization on adaptive radiation, ecology and life history.

## Conclusions

The newly born homodiploid 2nNCRC derived from the hybridization of *C. carpio* and *M. amblycephala* showed very similar phenotypic and genetic characteristics with the diploid *C. auratus* complex in nature. The very similar morphological characteristics and identical structures of the pharyngeal teeth between 2nNCRC and the *C. auratus* complex were totally different from those of *C. carpio* and *M. amblycephala*. The new structure of pharyngeal teeth suggested that 2nNCRC occupied a novel and suitable ecological niche from its parents, suggesting typical HHS in fish. Molecular phylogenetic analyses revealed an intraspecies relationship between 2nNCRC and the *C. auratus* complex. According to the reconstruction of ancestral areas and molecular data, the *C. auratus* complex lineage most likely originated from the Yangtze River basin in China during the Pliocene. The divergence pattern of the *C. auratus* complex in China was attributed to Pleistocene radiation, and the molecular data exactly coincided with the interglacial periods that occurred during the early and mid-Pleistocene in China. The diversification in the *Carassius* indicated that the *C. auratus* complex was impossible to derive from the recent common ancestor of *C. carassius* or *C. cuvieri*. HHS for 2nNCRC provides a good candidate speciation route for *C. auratus*, as the prerequisite condition of HHS and the hybridization between divergence lineages caused by the oscillation of the geologic climate during the Pliocene and Pleistocene could be satisfied in China.

## Materials and methods

### Samples and sequence data preparation

All of the 2nNCRC samples, including F_1_ to F_5_ generations used in this study, were cultured in ponds at the Protection Station of Polyploidy Fish, Hunan Normal University, and fed artificial feed. The F_1_ generation of 2nNCRC was denoted as 2nNCRC-F_1_, the F_2_ generation of 2nNCRC was the F_1_ self-crossed offspring and denoted as 2nNCRC-F_2_, and the naming procedure was followed for 2nNCRC-F_3_, 2nNCRC-F_4_ and 2nNCRC-F_5_. One specimen of each generation (F_1_ to F_3_) was collected, and a total of three mitochondrial genomes of 2nNCRC were sequenced to construct a phylogenetic tree based on two rRNAs and twelve protein-coding genes (ND6 was excluded) (the methods for DNA extraction, amplification, and sequencing are given in Additional file 1 in the Supplementary Information). To perform a comprehensive phylogenetic analysis, another 85 specimens of representative Cyprinidae fish (including 29 specimens of *Carassius*, 19 of *Cyprinus*, 14 of *Megalobrama* and 23 of other cyprinid fish) and 10 Catostomidae fish with complete mitochondrial genomes (both rRNAs and twelve protein-coding genes) were retrieved from GenBank (see Additional file 2 in Supplementary Information). In addition, a total of 831 cytb genes of the specimens (including three specimens in each generation of 2nNCRC-F_1_ to F_5_) in the genus *Carassius* widely distributed across Eurasia were also acquired from GenBank (see Additional file 3 in Supplementary Information) to obtain the phylogeographic structure of *Carassius* across Eurasia.

As very few nuclear DNA data are shared for *Carassius* and other Cyprinidae in GenBank, only HoxA2b was used to reconstruct the phylogenetic tree in present study. Two specimens of each generation (2nNCRC-F_1_ to F_3_) were sampled with two clones for each individual, and a total of 12 partial cds genes for HoxA2b were amplified (see Additional file 1 in Supplementary Information). In addition, the partial cds of HoxA2b for both parents (one for *M. amblycephala* and two for *C. carpio*) of 2nNCRC were also amplified, and 40 other specimens of Cyprinidae representing 21 species (see Additional file 4 in Supplementary Information) were obtained from GenBank in this study.

A previous study showed that the morphometrics of crucian carp-like fish (2nNCRC) were very similar to those of *C. auratus* in terms of barbel loss and a few lateral scales and even some measurable traits [13]. In this study, the morphological characteristics of other qualitative and quantitative traits were compared among the species of *Carassius* and 2nNCRC, as well as their parents (see Table S1 in Supplementary Information). To further test the morphological differences among 2nNCRC, *C. auratus*, *C. carpio* and *M. amblycephala*, geometric morphometrics was used to examine shape variation through principal component analysis (PCA) and canonical variance analysis (CVA) in MorphoJ v2.0 [82]. The left side of a specimen was placed on a moist foam mat at the base to maintain its natural configuration and photographed with a Nikon CoolPix 4500. Then, we digitized sixteen landmarks (see Fig. S1 in Supplementary Information) in each fish to capture the variation across the head and body shape, and the X and Y coordinates were recorded according to Zamani-Faradonbe et al. [83] by the software TpsDig2 [84]. For each specimen, the X and Y landmark coordinates were translated to the origin, rotated, scaled and superimposed by GPA (generalized Procrustes analysis) [85] in TpsRelwarp [86] to remove nonshape variations (such as location, orientation and scale). The specimen number of each species was more than 25 in this study. The CVA differentiates mutually exclusive groups by analyzing their relative positions in the morphospace and mapping shape differences onto the two first variates that explain the majority of the morphospace. This process is performed through the construction of a coordinate system called the canonical variates by determining the scores on those axes for all individuals, and they are referred to as eigenvalues [87].

Furthermore, pairwise genetic distances of inter- and intraspecific variation in the *Carassius* genus (see Table S5 in Supplementary Information) were calculated under the K2P model for the cytb dataset using MEGA 7 [88], as the cytb dataset is the most even of the datasets in GenBank.

### Phylogenetic analysis

All sequences were aligned with MAFFT [89] as implemented in Geneious 4.8.3. For the 98 mitochondrial genomes, the species of Catostomidae (see Additional file 2 in Supplementary Information) that shared a more recent common ancestry with Cyprinidae than with any other extant group of Cypriniform based on the pharyngeal feeding structures [90] were used as outgroups, and all alignments (twelve proteins and two rRNAs) were combined. We tested the 14 genes’ data for saturation using DAMBE v. 6.4.41 [91]. The test revealed an ISS value that was significantly lower than the ISS.c in all cases (*P* < 0.0000), indicating the suitability of the fourteen genes for phylogenetic analysis. The homogeneity of the fourteen genes was tested in *PAUP [92], and the *P* value was 0.69 (> 0.05). The 57 partial cds of HoxA2b (nuclear DNA) included 12 clones of 2nNCRC and 43 specimens of other Cyprinidae species, as well as the outgroup *Colossoma macropomum* and *Esox Lucius* (see Additional file 4 in Supplementary Information). The alignments (the conservative sequence after removing GAP) were also tested for saturation using DAMBE v. 6.4.41, and the ISS-value was significantly lower than the ISS.c value in all cases (*P* < 0.0000). To test our first hypothesis that 2nNCRC is *C. auratus*, ML and Bayesian trees using both the HoxA2b and mitochondrial genomes (incorporating the 14-gene data) were generated using RAxML v. 8.0 [93] with 1000 bootstrap replicates and MrBayes v. 3.2 [94], respectively. In the phylogenetic tree analysis, we determined the best-fitting substitution models for each gene fragment using Moldetest 3.7 [95], which are shown in Table S6 in the Supplementary Information. Mixing of the MCMC chains of the two independent runs was monitored with TRACER v. 1.7.1 [96], and the analysis was terminated after the average standard deviation of the split frequencies fell to less than 0.01. The first 25% of the sampled approximately 20 million generations were discarded as burn-in. The final trees were visualized in FIGTREE v. 1.4.4 [97].

To test our second hypothesis that several geographic lineages of *Carassius* endemic to specific regions of Eurasia exist, especially for *C. auratus* in China, we generated two haplotype networks using 831 cytb genes for all in Eurasia and 128 cytb for samples only in China and colored each haplotype by the geographic region from which it was collected (see Additional file 3 in Supplementary Information). Only the cytb gene was used in the haplotype network, as it had the most abundant data with population information for the five species of *Carassius* and is often used for population genetics in fish. The haplotype networks were constructed using network v. 10 (www.fluxus-engineering.com/sharenet.htm) and applying the median-joining and maximum parsimony options.

### Divergence time estimation

To test our third hypothesis that *C. auratus* originated from the hybridization between *C. carpio* and *M. amblycephala* in China, the precondition would need to be that TMRCA of both *Cyprinus* and *Megalobrama* had a much earlier divergence than that of the *C. auratus* complex, and all would have had to have a sympatric distribution in history. Furthermore, these lineages of the *C. auratus* complex distributed in Eurasia should belong to native lineages distributed in China. The divergence time was estimated using a molecular clock approach as implemented in BEAST. We used the combined data (98 specimens) including twelve proteins and two rRNAs in the phylogenetic tree and employed a (uncorrelated lognormal) relaxed clock, as a likelihood-ratio test (LRT) rejected the strict molecular clock hypothesis for the data (*P* < 0.01). As the lack of credible and exact fossil data can be found for the species in this study, we used a conservative approach by choosing three calibration points with normal distribution priors: the oldest fossil of *Plesiomyxocyprinus arratiae*, which is similar to *Myxocyprinus asiaticus*, was constrained from the middle Eocene or earlier, approximately 40–38 Mya [32], which is often used as the calibration point of *M. asiaticus* [30, 90]; the minimum age of Catostomidae is 60 Ma based on a catostomid fossil from the Paleocene [31]; and the timing of the drastic uplift of the Tibet Plateau occurred between 25 and 17 Mya [33] and was utilized as one calibration point for the Schizothoracinae as the species endemic to Qinghai-Tibet Plateau. The clade corresponding to each calibration point was constrained to be monophyletic. The GTR + I + G model was the best fit for the combined dataset (Table S6 in Supplementary Information). The ‘speciation: Yule process’ tree prior was used to construct the tree. We ran four independent runs for 20 million generations, logging trees every 2000 generations. Convergence was checked with TRACER v. 1.7.1. Posterior trees from the four runs were combined after removing the first 10% as burn-in in LogCombiner v. 2.5.2 (http://beast.bio.ed.ac.uk/logcombiner). The maximum credibility tree was created in TreeAnnotator v. 2.5.2 available in the BEAST package.

Furthermore, another divergence time was estimated using the cytb data. We used a reduced dataset of cytb: specimens for which two or more sequences from the same region were included. This approach resulted in 247 terminals – 237 representatives of the five species of *Carassius* and 13 outgroup sequences (*C. carpio*) from GenBank (see Additional file 5 in Supplementary Information). To minimize error, we used a conservative approach by employing calibration points from previous studies [24]. TMRCA of *C. carassius* in the northern and central-eastern European drainages and the Danubian catchment was constrained to 2.18–2.15 Mya. Another calibration point was the fossil of *Carassius* in the Pliocene epoch (5.3–2.6 Mya) in northern China (Yushe, Shanxi Province). The GTR + I + G model was also the best fit for the cytb dataset. The ‘speciation: Yule process’ tree prior was used to construct the tree. We ran four independent runs for 50 million generations, logging trees every 5000 generations. The other settings were same as those noted above.

### Reconstruction of ancestral areas

We further performed a biogeographic reconstruction of ancestral areas for species of *Carassius* using BioGeoBEARS [98] in RASP 4.02 [99] to determine whether overlapping distributions existed between *C. auratus* and other species of *Carassius*. The analyses were conducted on a fully resolved topology from the BEAST analysis containing 24 cytb sequences and five species of *Carassius* (see Additional file 6 in Supplementary Information). Seven major geographical areas were defined based on the worldwide distribution of *Carassius* according to its current distribution and Jeffries et al. [24]: (A) Europe, except for the southern Alpes Mountains and Danube River basin, (B) the southern Alpes Mountains and Danube River basin in Europe, (C) Western Asia, (D) Siberia in Russia and Mongolia, (E) China, (F) Southeast Asia, and (G) East Asia—Japan. To confirm that TMRCA of both *Cyprinus* and *Megalobrama* had a sympatric distribution in China, another biogeographic reconstruction of ancestral areas for *Cyprinus*, *Megalobrama* and *C. auratus* complex just distributed in China was conducted using the mitochondrial genome (twelve proteins and two rRNAs) of 26 specimens (the detailed sample location for each please see Additional file 7) representing 12 individuals of *Cyprinus*, 8 individuals of *Megalobrama*, and 6 individuals of *Carassius*. Six major geographical areas were defined based on their current distribution in China: (a) the Yangtze River basin, (b) the Pearl River basin, (c) the Yellow River basin, (d) the river basin of southeastern China, (e) the Amur River basin, and (f) the river basin of southwestern China.

All six models of geographic range evolution were compared in a likelihood framework (DEC, DEC + *j*, DIVALIKE, DIVALIKE + *j*, BAYAREALIKE and BAYAREALIKE + *j*). The best-fit model was assessed by comparing Akaike’s information criterion and likelihood-ratio tests, and DIVALIKE + *j* was chosen (see Table S7 in Supplementary Information). To account for phylogenetic uncertainty, 4000 post burn-in trees resulting from the BEAST analysis were integrated for inference. The maximum number of ancestral areas was set to four, as *C. auratus* and *C. carpio* can be widespread.

## Supplementary Information

**Additional file 1.** DNA extraction, PCR amplification, cloning and sequencing.

**Additional file 2.** Sequence information including the GenBank accession number, species name and ID for reconstructing the phylogenetic tree based on 98 mitochondrial genome.

**Additional file 3.** Sequence information including the GenBank accession number, species name, sampling location of 831 cytb for haplotype network.

**Additional file 4.** Sequence information including the GenBank accession number, species name and ID for reconstructing the phylogenetic tree based on HoxA2b gene.

**Additional file 5.** Sequence information including the GenBank accession number, species name, sampling location of 247 cytb for divergence time estimation.

**Additional file 6.** Sequence information including species name, GenBank accession number and sampling localities of 24 cytb for ancestral range reconstruction.

**Additional file 7. **Sequence information including species name, GenBank accession number and River system of 26 mitochondrial genome for ancestral range reconstruction of *Cyprinus*, *Megalobrama* and *C. auratus* complex just distributed in China.

**Additional file 8: Figure S1.** Positions of 16 landmarks superimposed on a photograph of fish.

**Additional file 9: Figure S2.** Ancestral range reconstruction for the *Carassius* across Eurasia using 24 sequence of cytb. The colors of the charts correspond to the most likely ancestral areas inferred, and the black color means the unknown area. Letters represent the biogeographic regions same with that in Fig. 3a. The blue curves in the distribution map of *Carassius* mean the river systems.

**Additional file 10: Table S1.** Morphological data of qualitative and quantitative traits including barbells, lateral scales, first gill rakers number, Spines and Soft-rays in Dorsal fin and Anal fin, as well as pharyngeal teeth formula of among 2nNCRC (F_1_-F_5_ generations), *C. auratus*, *C. carpio*, *M. amblycephala*, *C. gibelio*, *C. cuvieri*, and *C. langsdorfii*, and *C. carassius* (Kalous et al., 2007; Fishbase: https://www.fishbase.se/Nomenclature/ScientificNameSearchList.php?). **Table S2.** Principal component analysis on the variation of geometrical morphology among *C. auratus*, 2nNCRC, *C. carpio*, *M. amblycephala*, including the eigenvalues, percentage of variance and cumulative percentage of the first three principle components. **Table S3.** Canonical variate analysis on the variation of geometrical morphology among *C. auratus*, 2nNCRC, *C. carpio*, *M. amblycephala*, including the eigenvalues, percentage of variance and cumulative percentage of the three canonical variate axes. **Table S4.** Differences based on geometric morphometrics of fish shape among *C. auratus*, 2nNCRC, *C. carpio*, *M. amblycephala*. Mahalanobis (lower triangular) and Procrustes (upper triangular) distances computed from the Canonical Variate analysis, *P*-values for the significance of the interspecies distances were computed using permutation tests (10,000 replications); all *P* < 0.0001. **Table S5.** Pairwise genetic distance among groups including *C****.*** *auratus*, 2nNCRC, *C. cuvieri*, *C. langsdorfii*, *C. gibelio*, and *C. carassius* were calculated under the K2P model for the cytb dataset using MEGA v7.0 (Kumar et al., 2016), within group genetic distance are in bold. **Table S6.** Descriptive statistics for the fourteen genes alignments giving alignment length in base pairs (bp); model of sequence evolution for phylogenetic analysis. **Table S7.** Comparison of models used for BioGeoBEARS based on 24 cytb and 26 mtDNA; likelihood scores (LnL), number of parameters (numparams), dispersal rate (d), extinction rate (e), free parameter controlling the relative probability of founder-event speciation events at cladogenesis (j), corrected Akaike Information Criterion (AICc), and AICc model weights.

## Data Availability

Many genetic data were obtained through the GenBank (Additional file 2 and file 3 in Supplementary Information), and the new sequences data for 2nNCRC have been submitted in GenBank. The new datasets generated and analysed during the current study are available in the GenBank (https://www.ncbi.nlm.nih.gov/nuccore/?term=), and the accession number was OM240550-OM240576 (see Additional files 3 and 4 in Supplementary Information).
