# Supplementary material for: Phylogeographic relationships and the evolutionary history of the Carassius auratus complex with a newly born homodiploid raw fish (2nNCRC)
Source: BMC Genomics. 2022 Mar 28;23:242. doi: 10.1186/s12864-022-08468-x (PMC8962218; doi:10.1186/s12864-022-08468-x)
Supplement: Supplementary file 1 — Additional file 1. DNA extraction, PCR amplification, cloning and sequencing. [file 12864_2022_8468_MOESM1_ESM.docx]

**DNA extraction, PCR amplification, cloning and sequencing**

Total genomic DNA from the peripheral blood cells of fish (*C. carpio*, *M. amblycephala*, *C. auratus*, *C. cuvieri* and 2nNCRC) was extracted by routine approaches (Sambrook and Russell, 1989). The highly conserved and newly designed PCR primers (You et al., 2014) were used to amplify up to 22 contiguous and overlapping fragments of the complete mitochondria genome sequences in 2nNCRC (F_1_ to F_3_). Furthermore, Cytb genes of fifteen specimens (three in each of 2nNCRC-F_1_ to F_5_) were amplified by PCR using the forward and reverse primers L14736-Glu and H15923-Thru (Takada et al., 2010). The PCRs were performed in a volume of 50 μL with approximately 10-30 ng of genomic DNA, 1.5 mM MgCl2, 250 μM dNTP, 0.4 μM each primer, and 1.25 U of Taq polymerase (TaKaRa, Dalian, China). The thermal program consisted of an initial denaturation step at 94°C for 5 min, followed by 30 cycles of 94°C for 35 s, 50-60°C for 60 s, and 72°C for 60-150 s and a final extension step at 72°C for 10 min (You et al., 2014). A majority of the PCR products were directly sequenced, and some fragments that were difficult to sequence using PCR products were cloned into the pMD18-T vector (TaKaRa, Dalian, China), the plasmids were transformed into *E. coli* DH5a and purified (Guo et al., 2006). At least three clones from each PCR product were sequenced with vector-specific primers using the primer walking method on an ABI 3730XL automatic sequencer (ABI PRISM 3730, Applied Biosystems, CA, USA). The sequence homology and variation among the fragments amplified were analysed using BioEdit (Hall, 1999) and the DNAstar 5.0 software package (DNAstar Inc.).

The primers for the amplification of HoxA2b gene in 2nNCRC, *C. carpio* and *M. amblycephala* included the forward primer [CTCGCTGAGTGCCTGACAT] and reverse primer [GAGAAAACRTTGAAG TCCTGYAAA]. PCR was performed in a total volume of 50 μL with approximately 10–30 ng of genomic DNA, 1.5 mM MgCl_2_, 250 μM dNTPs, each primer at 0.4 μM, and 1.25 U of Taq polymerase (TaKaRa, Dalian, China). The thermal program consisted of an initial denaturation step of 94°C for 5 min, followed by 35 cycles of 94°C for30 s, 50–60°C for 30 s, and 72°C for 1–3 min, with a final extension step of 72 °C for 7 min. A majority of the PCR products were directly sequenced, and some fragments that were difficult to sequence using the PCR products were cloned into the pMD18-T vector (TaKaRa, Dalian, China). The plasmids were transformed into E. coli DH5a cells and purified. To increase the probability of detecting duplicated paralogs and to circumvent errors owing to PCR, two clones of each gene from each sample were sequenced with vector-specific primers using the primer walking method on an ABI 3730XL automatic sequencer (ABI PRISM3730, Applied Biosystems, CA, USA). The obtained sequences were screened for HoxA2b gene fragments using BLAST (http://www.ncbi.nlm.nih.gov) searches.

Sambrook, J., and Russell, D.W. (1989). *Molecular cloning: a laboratory manual* (Vol. 3). New York, NY: Cold spring harbor laboratory press.

Takada, M., Tachihara, K., Kon, T., Yamamtoto, G., Lguchi, K., and Miya, M. (2010). Biogeography and evolution of the *Carassius auratus*-complex in East Asia. BMC Evolutionary Biology 10, 7.

You, C.P., Zhao, R.R., Hu, J., Liu, S., Tao, M., Zhang, C., Chen, Y.B., Qin, Q.B., Xiao, J., and Duan, W. (2014). Inheritance of the complete mitochondrial genomes in three different ploidy fishes. Current Molecular Medicine 14, 1322-1330.

Guo, X., Liu, S., and Liu, Y. (2006). Evidence for recombination of mitochondrial DNA in triploid crucian carp. Genetics 172, 1745-1749.

Hall, T.A. (1999). BioEdit: a user-friendly biological sequence alignment editor and analysis program for Windows 95/98/NT[C]//. Nucleic acids symposium series. [London]: Information.
