# Supplementary figures and images for: Phylogeographic relationships and the evolutionary history of the Carassius auratus complex with a newly born homodiploid raw fish (2nNCRC)
Source: BMC Genomics. 2022 Mar 28;23:242. doi: 10.1186/s12864-022-08468-x (PMC8962218; doi:10.1186/s12864-022-08468-x)

## Slide 1
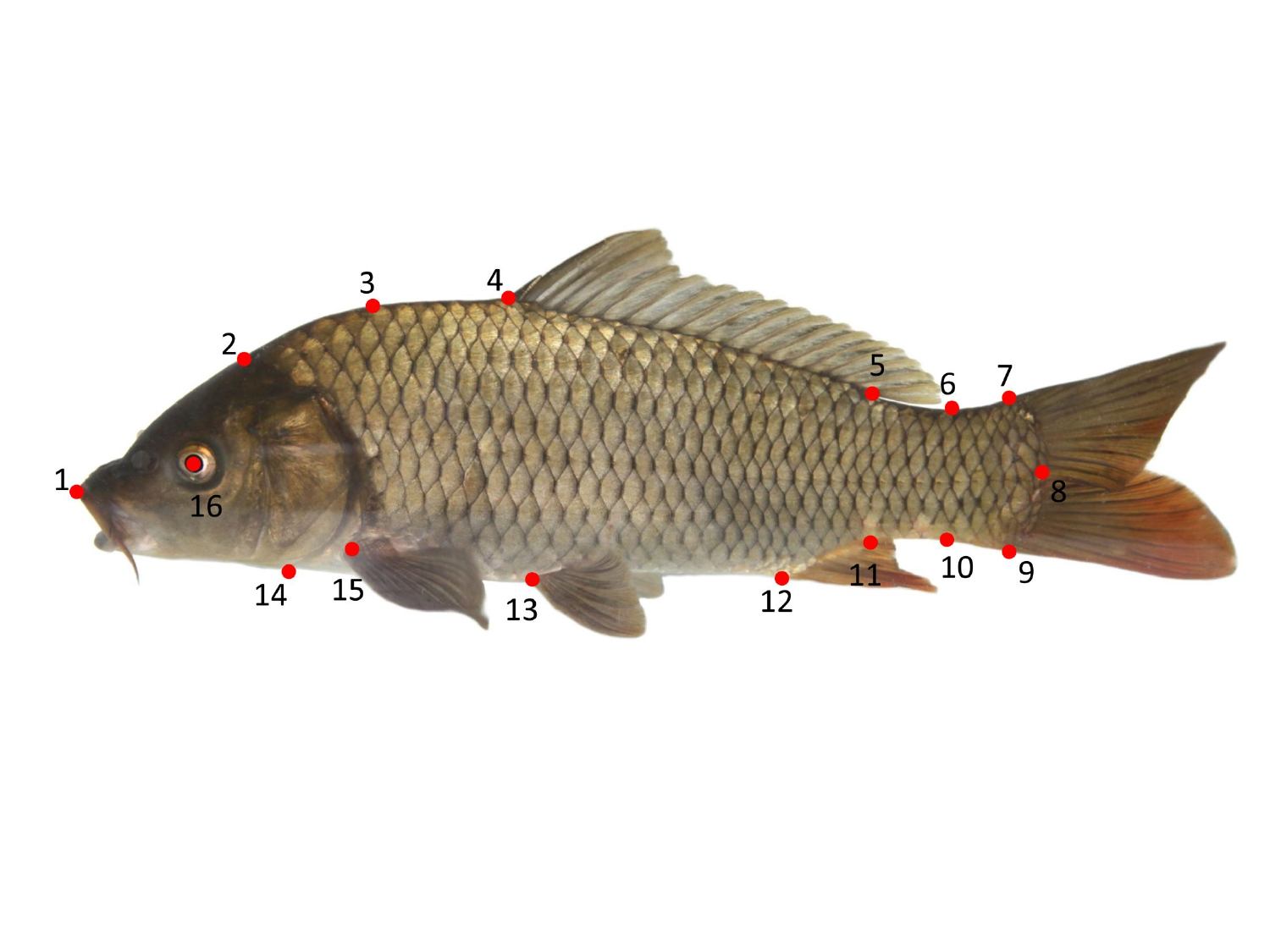

Supplement: Supplementary file 8 — Additional file 8: Figure S1. Positions of 16 landmarks superimposed on a photograph of fish. [file 12864_2022_8468_MOESM8_ESM.pptx]

## Slide 1
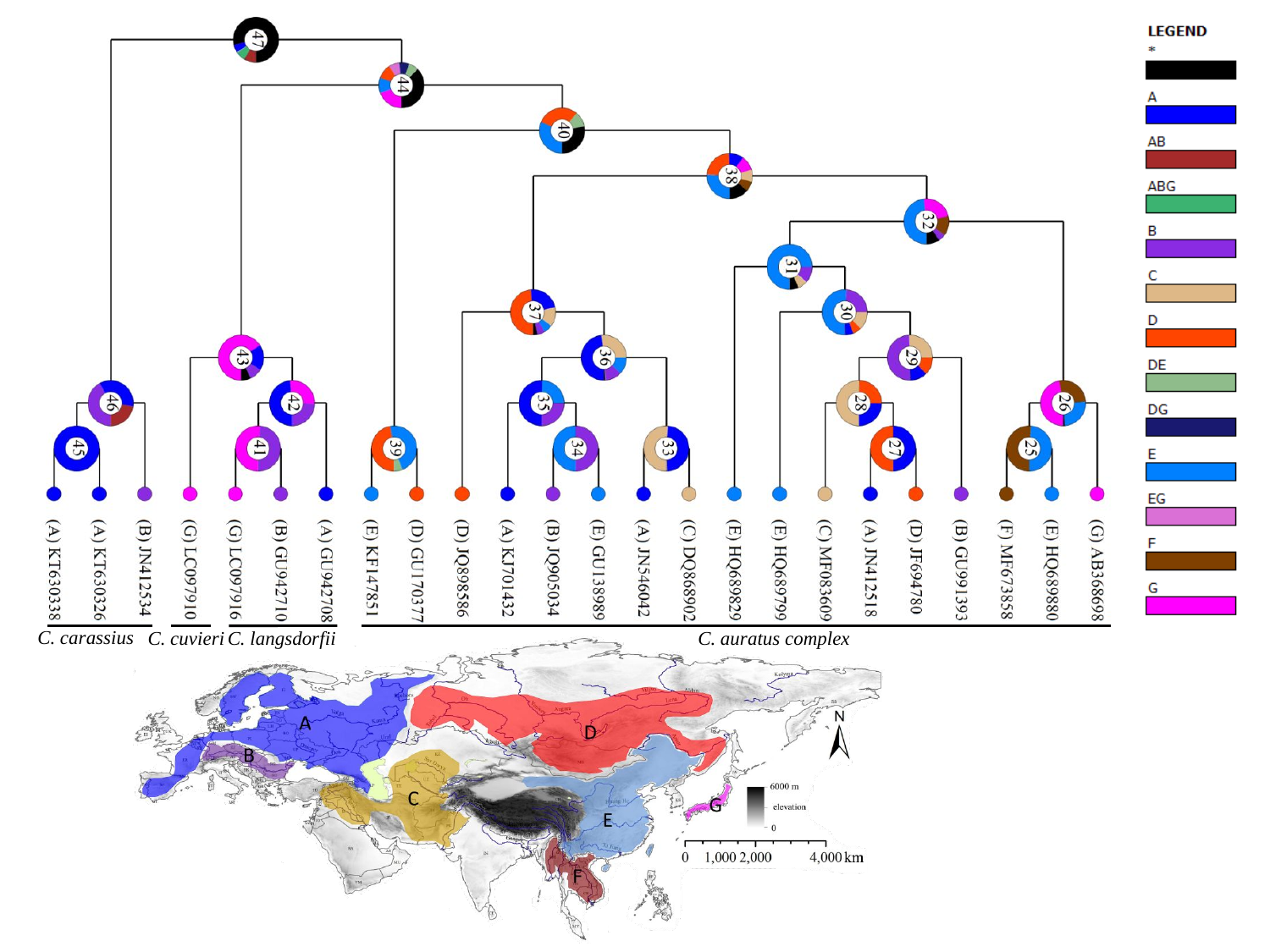

C. carassius
C. langsdorfii
C. auratus complex
C. cuvieri

Supplement: Supplementary file 9 — Additional file 9: Figure S2. Ancestral range reconstruction for the Carassius across Eurasia using 24 sequence of cytb. The colors of the charts correspond to the most likely ancestral areas inferred, and the black color means the unknown area. Letters represent the biogeographic regions same with that in Fig. 3a. The blue curves in the distribution map of Carassius mean the river systems. [file 12864_2022_8468_MOESM9_ESM.pptx]
