# Supplementary material for: Phylogeographic relationships and the evolutionary history of the Carassius auratus complex with a newly born homodiploid raw fish (2nNCRC)
Source: BMC Genomics. 2022 Mar 28;23:242. doi: 10.1186/s12864-022-08468-x (PMC8962218; doi:10.1186/s12864-022-08468-x)
Supplement: Supplementary file 10 — Additional file 10: Table S1. Morphological data of qualitative and quantitative traits including barbells, lateral scales, first gill rakers number, Spines and Soft-rays in Dorsal fin and Anal fin, as well as pharyngeal teeth formula of among 2nNCRC (F1-F5 generations), C. auratus, C. carpio, M. amblycephala, C. gibelio, C. cuvieri, and C. langsdorfii, and C. carassius (Kalous et al., 2007; Fishbase: https://www.fishbase.se/Nomenclature/ScientificNameSearchList.php?). Table S2. Principal component analysis on the variation of geometrical morphology among C. auratus, 2nNCRC, C. carpio, M. amblycephala, including the eigenvalues, percentage of variance and cumulative percentage of the first three principle components. Table S3. Canonical variate analysis on the variation of geometrical morphology among C. auratus, 2nNCRC, C. carpio, M. amblycephala, including the eigenvalues, percentage of variance and cumulative percentage of the three canonical variate axes. Table S4. Differences based on geometric morphometrics of fish shape among C. auratus, 2nNCRC, C. carpio, M. amblycephala. Mahalanobis (lower triangular) and Procrustes (upper triangular) distances computed from the Canonical Variate analysis, P-values for the significance of the interspecies distances were computed using permutation tests (10,000 replications); all P < 0.0001. Table S5. Pairwise genetic distance among groups including C. auratus, 2nNCRC, C. cuvieri, C. langsdorfii, C. gibelio, and C. carassius were calculated under the K2P model for the cytb dataset using MEGA v7.0 (Kumar et al., 2016), within group genetic distance are in bold. Table S6. Descriptive statistics for the fourteen genes alignments giving alignment length in base pairs (bp); model of sequence evolution for phylogenetic analysis. Table S7. Comparison of models used for BioGeoBEARS based on 24 cytb and 26 mtDNA; likelihood scores (LnL), number of parameters (numparams), dispersal rate (d), extinction rate (e), free parameter control [file 12864_2022_8468_MOESM10_ESM.docx]

**Table S1** Morphological data of qualitative and quantitative traits including barbells, lateral scales, first gill rakers number, Spines and Soft-rays in Dorsal fin and Anal fin, as well as pharyngeal teeth formula of among 2nNCRC (F_1_-F_5_ generations), *C. auratus*, *C. carpio*, *M. amblycephala*, *C. gibelio*, *C. cuvieri*, and *C. langsdorfii*, and *C. carassius* (Kalous et al., 2007; Fishbase: https://www.fishbase.se/Nomenclature/ScientificNameSearchList.php?)

|  | Barbells | Lateral  scales | First gill rakers  number | Spines and Soft-rays in  Dorsal fin | Spines and Soft-rays in  Anal fin | Pharyngeal teeth  formula |
| --- | --- | --- | --- | --- | --- | --- |
| 2nNCRC | no | 29 – 32 | 49 – 53 | iii, 15 - 18 | iii, 5 - 6 | 4 / 4 |
| *C. auratus* | no | 21 – 36 | 37 – 54 | iii – iv, 14 – 20 | iii, 4 – 7 | 4 / 4 |
| *C. gibelio* | no | 28 – 32 | 49 – 54 | ii – iii, 16 - 19 | iii, 5 -6 | 4 / 4 |
| *C. cuvieri* | no | 29 – 33 | 92 – 128 | iv, 15 - 18 | iii, 4 - 5 | 4 / 4 |
| *C. langsdorfii* | no | 28–31 | 41–57 | iv, 15 - 18 | ii, 5 - 8 | 4 / 4 |
| *C. carassius* | no | 21 – 38 | 23 – 33 | iii – iv, 14 - 25 | ii – iii, 5 - 8 | 4 / 4 |
| *C. carpio* | two pairs | 33 – 39 | 27 – 28 | iii - iv, 17 - 23 | ii - iii, 5 - 6 | 1, 1, 3 / 3, 1, 1 |
| *M. amblycephala* | no | 50 – 60 | 13 – 15 | iii, 7 | iii, 27 - 32 | 2, 4, 5 / 4, 4, 2 |

**Table S2** Principal component analysis on the variation of geometrical morphology among *C. auratus*, 2nNCRC, *C. carpio*, *M. amblycephala*, including the eigenvalues, percentage of variance and cumulative percentage of the first three principle components.

| Principle components | Eigenvalues | % Variance | Cumulative % Variance |
| --- | --- | --- | --- |
| PC1 | 0.00654765 | 77.093 | 77.09 |
| PC2 | 0.00094819 | 11.164 | 88.26 |
| PC3 | 0.00051434 | 6.056 | 94.31 |

**Table S3** Canonical variate analysis on the variation of geometrical morphology among *C. auratus*, 2nNCRC, *C. carpio*, *M. amblycephala*, including the eigenvalues, percentage of variance and cumulative percentage of the three canonical variate axes.

|  | Eigenvalues | % Variance | Cumulative % Variance |
| --- | --- | --- | --- |
| CV1 | 682.10391757 | 88.711 | 88.71 |
| CV2 | 70.95726293 | 9.228 | 97.94 |
| CV3 | 15.84212110 | 2.060 | 100.00 |

**Table S4** Differences based on geometric morphometrics of fish shape among *C. auratus*, 2nNCRC, *C. carpio*, *M. amblycephala*. Mahalanobis distances computed from the Canonical Variate analysis，*P*-values for the significance of the interspecies distances were computed using permutation tests (10000 replications); all *P* < 0.0001.

|  | *C. auratus* | 2nNCRC | *C. carpio* | *M. amblycephala* |
| --- | --- | --- | --- | --- |
| *C. auratus* |  |  |  |  |
| 2nNCRC | 12.2338 |  |  |  |
| *C. carpio* | 22.4184 | 20.0354 |  |  |
| *M. amblycephala* | 57.2515 | 60.8495 | 65.5496 |  |

**Table S5** Pairwise genetic distance among groups including *C****.*** *auratus*, 2nNCRC, *C. cuvieri*, *C.* *langsdorfii*, *C. gibelio*, and *C. carassius* were calculated under the K2P model for the cytb dataset using MEGA v7.0 (Kumar et al., 2016), within group genetic distance are in bold.

| Species | *C. auratus* | *C. cuvieri* | 2nNCRC | *C. carassius* | *C. gibelio* | *C. langsdorfii* |
| --- | --- | --- | --- | --- | --- | --- |
| *C. auratus* | **0.017** |  |  |  |  |  |
| *C. cuvieri* | 0.073 | **0.002** |  |  |  |  |
| 2nNCRC | 0.018 | 0.075 | **0.008** |  |  |  |
| *C. carassius* | 0.099 | 0.106 | 0.101 | **0.002** |  |  |
| *C. gibelio* | 0.019 | 0.073 | 0.018 | 0.098 | **0.002** |  |
| *C. langsdorfii* | 0.07 | 0.071 | 0.072 | 0.099 | 0.066 | **0.016** |

**Table S6** Descriptive statistics for the fourteen genes alignments giving alignment length in base pairs (bp); model of sequence evolution for phylogenetic analysis.

| Alignment | Length (bp) | Model | Invariable sites (I) | Gamma (α) |
| --- | --- | --- | --- | --- |
| *12S rRNA* | 977 | GTR+I+G | 0.5068 | 0.6599 |
| *16S rRNA* | 1733 | GTR+I+G | 0.4551 | 0.6111 |
| *ND1* | 976 | TrN+I+G | 0.5060 | 1.0308 |
| *ND2* | 1050 | TIM+I+G | 0.4231 | 1.1308 |
| *COI* | 1552 | K81uf+I+G | 0.5897 | 1.1931 |
| *COII* | 691 | HKY+I+G | 0.4646 | 0.8819 |
| *ATP8* | 168 | HKY+G | 0 | 0.6220 |
| *ATP6* | 684 | TrN+I+G | 0.4141 | 0.8083 |
| *COIII* | 786 | HKY+I+G | 0.5395 | 0.9274 |
| *ND3* | 351 | TrN+I+G | 0.4995 | 1.5347 |
| *ND4L* | 297 | TrN+G | 0 | 0.1856 |
| *ND4* | 1383 | TrN+I+G | 0.4888 | 1.1563 |
| *ND5* | 1841 | TrN+I+G | 0.4034 | 1.0557 |
| *Cytb* | 1145 | GTR+I+G | 0.5336 | 1.1054 |
| Concatenated | 13634 | GTR+I+G | 0.5022 | 0.9711 |

**Table S7** Comparison of models used for BioGeoBEARS based on 24 cytb and 26 mtDNA; likelihood scores (LnL), number of parameters (numparams), dispersal rate (d), extinction rate (e), free parameter controlling the relative probability of founder-event speciation events at cladogenesis (j), corrected Akaike Information Criterion (AICc), and AICc model weights.

| Data | Models | LnL | numparams | d | e | *j* | AICc | AICc_wt |
| --- | --- | --- | --- | --- | --- | --- | --- | --- |
| 24 sequences of cytb | DEC | -99.52 | 2 | 0.010 | 0.010 | 0 | 203.6 | 1.2E-21 |
|  | DEC + *j* | -51.2 | 3 | 1.0E-12 | 7.2E-12 | 0.31 | 109.6 | 0.32 |
|  | DIVALIKE | -89.9 | 2 | 0.010 | 0.010 | 0 | 184.4 | 1.9E-17 |
|  | DIVALIKE + *j* | -50.57 | 3 | 1.0E-12 | 1.0E-12 | 0.24 | 108.3 | 0.60 |
|  | BAYAREALIKE | -118.4 | 2 | 0.81 | 2.89 | 0 | 241.5 | 7.4E-30 |
|  | BAYAREALIKE + *j* | -52.54 | 3 | 1.0E-07 | 0.029 | 0.34 | 112.3 | 0.084 |
|  |  |  |  |  |  |  |  |  |
| 26 mtDNA | DEC | -81.99 | 2 | 0.018 | 0.01 | 0 | 168.5 | 8.60E-16 |
|  | DEC + *j* | -47.12 | 3 | 1.00E-12 | 0.0004 | 0.6 | 101.3 | 0.33 |
|  | DIVALIKE | -76.26 | 2 | 0.028 | 0.025 | 0 | 157 | 2.70E-13 |
|  | DIVALIKE + *j* | -46.67 | 3 | 1.00E-12 | 1.00E-12 | 0.44 | 100.4 | 0.52 |
|  | BAYAREALIKE | -124.8 | 2 | 0.16 | 0.49 | 0 | 254.1 | 2.30E-34 |
|  | BAYAREALIKE + *j* | -47.93 | 3 | 1.00E-07 | 0.0059 | 0.79 | 103 | 0.15 |

Kalous L, Slechtova VJ, Bohlen J, Petrtyl M, Svatora M. First European record of *Carassius langsdorfii* from the Elbe basin. J Fish Biol. 2007;270:132-138.

Kumar S, Stecher G, Tamura K. MEGA7: Molecular Evolutionary Genetics Analysis Version 7.0 for Bigger Datasets. Mol Biol Evol. 2016;33:1870-1874.
